# The implications of crustal architecture and transcrustal upflow zones on the metal endowment of a world-class mineral district

**DOI:** 10.1038/s41598-022-18836-y

**Published:** 2022-08-29

**Authors:** Taus R. C. Jørgensen, Harold L. Gibson, Eric A. Roots, Rajesh Vayavur, Graham J. Hill, David B. Snyder, Mostafa Naghizadeh

**Affiliations:** 1 Mineral Exploration Research Centre, Harquail School of Earth Sciences, Laurentian University, Sudbury, Canada; 2 Geological Survey of Canada, Ottawa, Canada; 3 Institute of Geophysics, Czech Academy of Science, Prague, Czech Republic

**Keywords:** Economic geology, Geology, Structural geology, Solid Earth sciences, Geophysics

## Abstract

Earth’s mineral deposits show a non-uniform spatial distribution from the craton-scale, to the scale of individual mineral districts. Although this pattern of differential metal endowment is underpinned by lithospheric-scale processes the geological features that cause clustering of deposits remains enigmatic. The integration of geological and geophysical (seismic, gravity, and magnetotelluric) features has produced the first whole-of-crust image through an iconic Neoarchean volcanic complex and mineral district in the Abitibi Greenstone Belt, Superior Province, Canada. Observations indicate an asymmetry in surface geology, structure, and crustal architecture that defines deep transcrustal magmatic-hydrothermal upflow zones and the limits of the Noranda District ore system. Here, extreme volcanogenic massive sulfide (VMS) endowment is confined to a smaller area adjacent to an ancestral transcrustal structure interpreted to have localized and optimized magmatic and ore forming processes. Although lithospheric-scale evolutionary processes might act as the fundamental control on metal endowment, the new crustal reconstruction explains the clustering of deposits on both belt and district scales. The results highlight a strong magmatic control on metal and in particular Au endowment in VMS systems. Overprinting by clusters of ca. 30 Ma younger orogenic Au deposits suggest the ore systems accessed an upper lithospheric mantle enriched in Au and metals.

## Introduction

**Figure 1:**
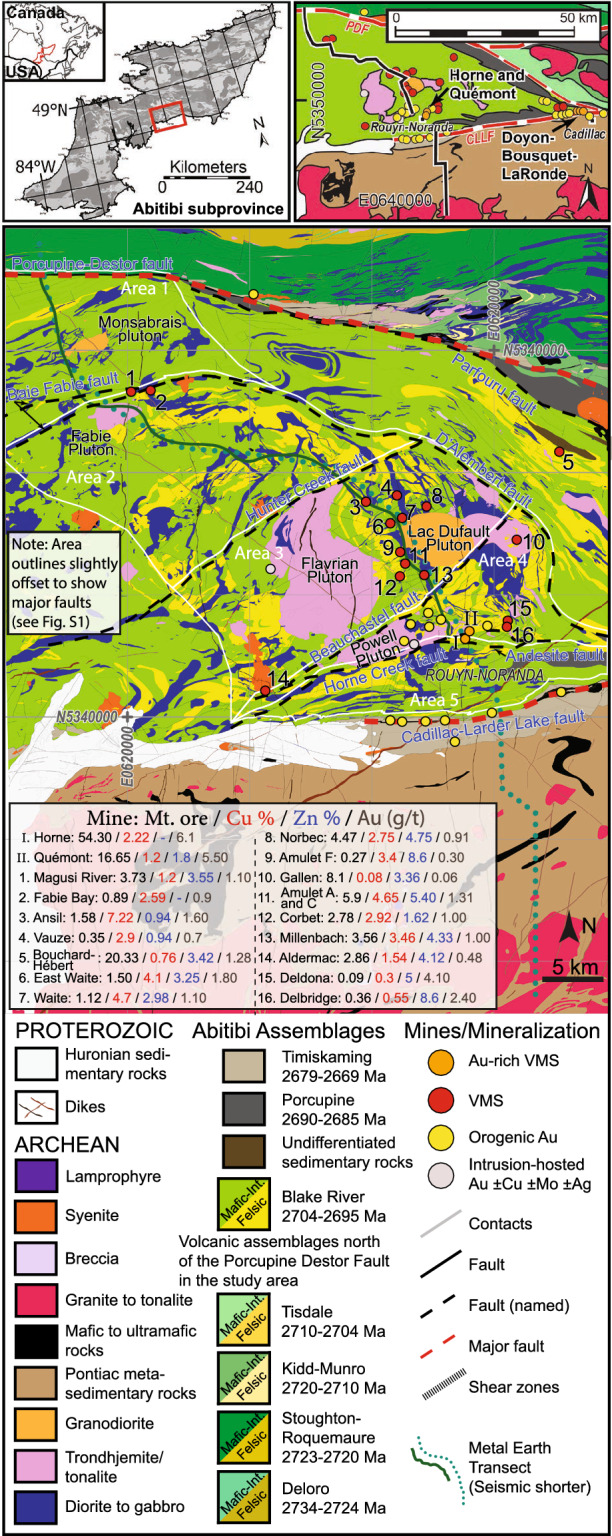
Geological map of the Noranda District with insets showing the study area (top left) and Doyon-Bousquet-LaRonde District (top right) locations. The maps in this Figure were generated in ArcGIS (≥ v. 10.5), Adobe Photoshop (≥ v. CC2019 20.0), and Adobe Illustrator (≥ v. CC2018 22.0.0) using open data downloaded from Système d'information géominière of Québec (SIGÉOM): https://sigeom.mines.gouv.qc.ca/signet/classes/I1102_aLaCarte?l=a#.

Lithospheric-scale crustal growth processes are considered first-order controls on metal source, transport, and concentration^1–3^. However, the crustal architecture and geological features that characterize mineral districts and the processes responsible for district-scale metal endowment are poorly constrained^4^. Accordingly, we have compiled and assessed geological data, and conducted seismic, gravity, and magnetotelluric (MT) surveys along a transect through the Neoarchean Noranda District, Superior Province (Quebec, Canada; Fig. 1). The Noranda District hosts ~ 20 VMS deposits (~ 130 Mt ore), ~ 19 orogenic Au deposits, and minor synvolcanic intrusion-hosted Cu–Mo ± Au ± Ag mineralization^5^. Considering the entire Superior craton contains 115 known VMS deposits, and the Archean Yilgarn and Pilbara cratons contains 22 combined^6^, a whole-of-crust analyses of the Noranda District provides the opportunity to identify belt and district-scale features and processes that influenced metal endowment. Although VMS deposits and districts share common features and ore forming processes^7,8^ we link the extreme base and precious metal endowment of the Noranda District and the formation of the largest Au-rich volcanogenic massive sulfide deposit ever discovered (Horne deposit: ~ 325 t Au) to distinctive crustal-scale features.

A quantitative examination of the spatial relationship between mineral deposits and lithospheric conductivity on a global scale, showed a strong correlation between orogenic gold deposits and mid-crustal conductors, whereas VMS deposits showed a weak association with upper mantle conductors^9^. However, orogenic gold deposits have been shown to be spatially associated with the borders of magmatic centres and rift faults also spatially connected to VMS mineralization despite occurring hundreds of millions of years later^10^. Such associations point to a primary crustal architecture responsible for focusing both VMS and later gold mineralization^10^. Anchored in the geological and geophysical integration presented in this study, we explore the possibility that localized lithospheric mantle-scale enrichment resulting from focused, long-lived, sustained magmatic and tectonic processes may be responsible for the endowment of the Noranda District. This pertains to VMS endowment, VMS Au-enrichment, and subsequent localization of orogenic Au deposits during later deformation events. Thus, VMS Au-enrichment may be a proxy for early upper lithospheric mantle Au and base metal enrichment^11^. Identification of the geodynamic processes leading to the extreme metal endowment that characterizes the Noranda District may explain the variable metal endowment in greenstone belts globally.

### Geological setting

The VMS-hosting, bimodal-mafic Noranda volcanic centre belongs to the 2704–2695 Ma Blake River assemblage of the Abitibi subprovince, southeastern Superior Province^12^ (Fig. 1). The Cadillac-Larder Lake fault (CLLF) dissect the Abitibi subprovince and juxtaposes the Noranda volcanic centre against the ~ 2682 Ma Pontiac subprovince and ~ 2672–2665 Ma Timiskaming assemblage to the south and the Porcupine-Destor fault (PDF) juxtaposes the 2723–2720 Ma Stoughton-Roquemaure assemblage to the north^13^ (Fig. 1). Both faults are auriferous transcrustal structures (100 s of km long) with a prolonged and complex kinematic history^14^. The Andesite, Horne Creek, Powell, Beauchastel, Hunter Creek and Baie Fabie faults are major, subvertical synvolcanic faults (Fig. 1) that dissect the Noranda District into distinct fault blocks^5,15^ (Fig. 1, Supplementary Fig. S1). The large, sill-like, Flavrian and Powell, and smaller Fabie subvolcanic tonalite-trondhjemite plutons (ca. 2701 Ma) define magmatic centres within the Noranda volcanic complex^16^. See Supplementary Appendix DR1 for an expanded geological setting.

## Results

### Geological features

**Figure 2:**
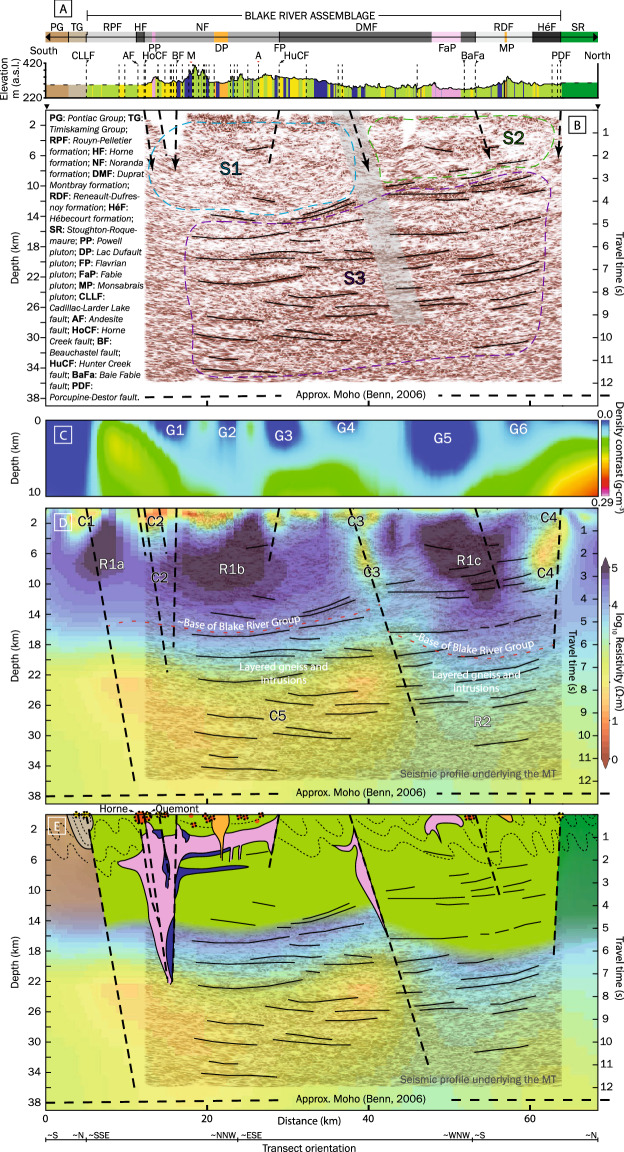
Geological (**A**; see Fig. 1 legend), seismic (**B**), density (**C**), and MT with interpreted solid black subhorizontal reflectors and dashed black subvertical faults (**D**) sections along the Metal Earth transect (Fig. 1). (**E**) Interpreted section showing VMS and orogenic Au deposits as red and yellow circles (on transect), respectively, with black dotted outline (projected). S1-3, G1-6, C1-5, and R1-2 correspond to features of interest discussed in the text. The Figure was compiled in Adobe Illustrator (≥ v. CC2018 22.0.0) with the geological section in panel **A**) generated using open data downloaded from Système d'information géominière of Québec (SIGÉOM): https://sigeom.mines.gouv.qc.ca/signet/classes/I1102_aLaCarte?l=a#, the seismic section in panel B was generated using the open-source seismic processing package Seismic Un*x: https://github.com/JohnWStockwellJr/SeisUnix, the density section in panel C was generated using Emerson Paradigm software SKUA-GOCAD-17: https://www.pdgm.com/products/skua-gocad, and the MT section in panel D was generated using an open-source collection of python projects for manipulation and visualization of MT data: https://github.com/eroots/pymt.

Areas 1–5 largely correspond to the distinct structural blocks with Areas 1–2 comprising the Hunter block, Area 3 the Flavrian block, Area 4 the Powell and Horne blocks, and Area 5 the Rouyn-Pelletier block (Fig. 1, Supplementary Fig. S1). The geological features are not equally distributed between the five areas (Figs. 1, 2A, Supplementary Fig. S1; Supplementary Tables S1, S2). Areas 3 and 4 are characterized by higher metal content, density of structures, and proportion of felsic volcanic rocks and synvolcanic TTG intrusions, with Area 4 having the largest deposits, highest Au grade and number of ounces (14 Moz Au).

### Geophysical features

The seismic survey was acquired along a ~ 50 km transect using vibroseis sources and vertical-component wireless geophones with source and receiver spacing of 50 m and 25 m, respectively. The data processing workflow was focused on appropriate binning of the crooked survey lines, robust static solutions, detailed velocity analysis, minimal trace smoothing, and high-resolution imaging using a pre-stack time migration algorithm^17^. Low seismic reflectivity extends from the surface to ~ 14–16 km depth across the southern part of the profile (S1), compared to ~ 6–8 km depth in the north (S2; Fig. 2B). Below this, the majority of prominent reflectors are subhorizontal to shallowly south dipping, continuous for ~ 2–20 km and are observed to the base of the section (S3), but are most pronounced in the ~ 14–27 km range. The distinct break in reflectors near the middle of S3 (most pronounced at ~ 10 km depth) coincides with two minor faults (Fig. 2B).

The gravity survey was conducted along the seismic transect with stations approximately every 300 m with side road stations up to 1 km from the transect. This dataset was integrated with existing regional data compiled by the Geological Survey of Canada (http://gdrdap.agg.nrcan.gc.ca/) and used to construct a 3D density model via 3D inversion^18,19^. The density cross-section was extracted from the final inverted 3D density model to match the seismic transect (Fig. 2C). An isosurface was extracted using the density contrast value of 0.07 g/cm^3^ (Fig. S2). At the southern margin of the density section, the Pontiac sedimentary rocks are defined by a pronounced low density anomaly that abruptly changes at the CLLF (Fig. 2A, C). North of the fault, the Noranda District is marked by six distinct density lows (G1-6) which, except for G4, coincide with the location of known subvolcanic intrusions. The correlation between low-density anomalies and synvolcanic TTG intrusions are equally observed in the 3D high-density region isosurface, where the largest low-density area in the Noranda District correspond to the location of the Flavrian and Powell synvolcanic intrusions (Supplementary Fig. S2). The Fabie pluton corresponds to a relatively large low-density anomaly on the density profile (G5) but only a subtle feature in the 3D model; the orientation of the transect parallel to the low-density volume resulted in an oversized profile anomaly (Supplementary Fig. S2). North of the PDF the density increases.

**Figure 3:**
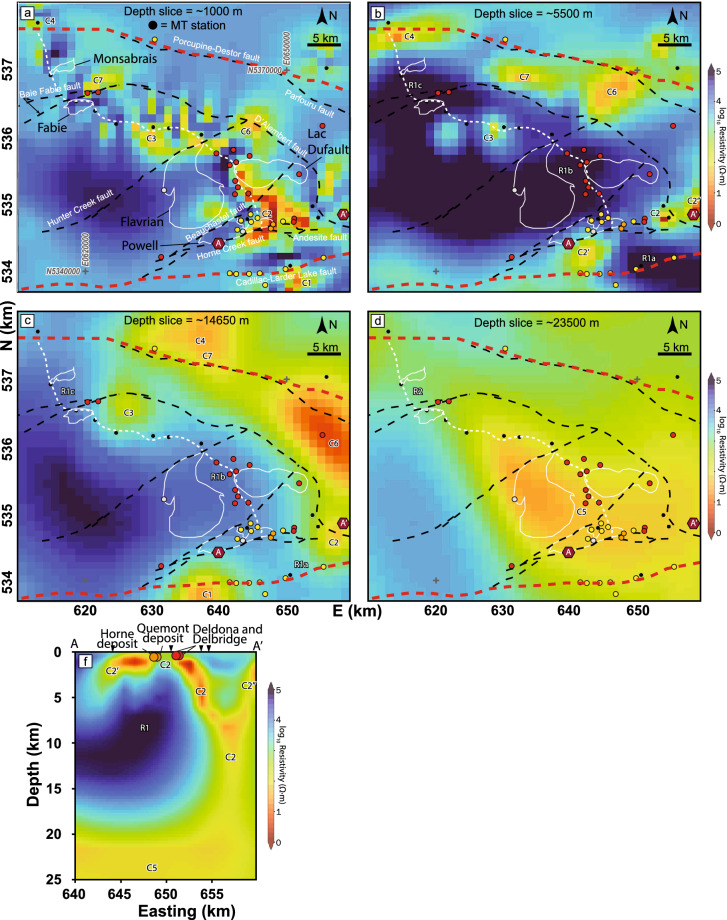
MT depth slices (**a**–**d**) and a longitudinal section (A-A’) along the Horne Creek Fault (**f**). White stippled line is the seismic transect, white solid lines are pluton outlines, black and red stippled lines are major faults, deposits markings follow Fig. 1. C1-7 indicates significant low-resistivity features (see text). This Figure was compiled in Adobe Illustrator (≥ v. CC2018 22.0.0) with individual panels generated using an open-source collection of python projects for manipulation and visualization of MT data: https://github.com/eroots/pymt.

**Figure 4:**
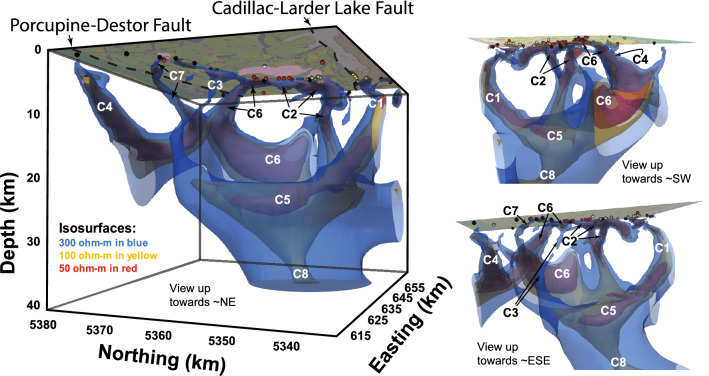
3D MT model with surface geology (see Fig. 1). C1-8 indicate significant low-resistivity features (see text). The two panels on the right are different perspectives of the same 3D model to help visualize the connectivity of many of the low-resistivity features. This Figure was compiled in Adobe Illustrator (≥ v. CC2018 22.0.0) with individual panels created in the open-source ParaView application: https://www.paraview.org/.

The MT model of the Noranda District is a subset of a larger, recently published resistivity model of the southeastern Superior region^20^. The larger model considers data from 112 broadband (~ 10^–2^–10^3^ s bandwidth) MT stations collected during the recent Metal Earth project^21^, and the Lithoprobe and Discovery Abitibi surveys^22^. Stations in the larger model are distributed along four approximately north–south transects with a station spacing of ~ 5 km, with some off-transect stations for the purpose of providing additional 3D coverage. Inversion of the data was done using the HexMT 3D finite element inversion algorithm^23,24^. A depth section was extracted from the 3D resistivity volume following the MT station distribution in the Noranda District, which is roughly coincident with the seismic, gravity, and geological transects (Fig. 2D). Intermittent near-surface (~ 1–2 km) low-resistivity (~ 10–100 Ω·m) areas coincide with the CLLF (C1), Horne Creek fault (C2), minor faults (C3), and the Porcupine-Destor fault (C4) (Fig. 2D). Otherwise, the upper crust is characterized by a relatively uniform high-resistivity (R1 > 1000 Ω·m) signature that extends down to ~ 14 km. The transition to a lower resistivity regime (≤ 300 Ω·m) coincides with the brittle-ductile transition zone (~ 10–15 km^25^). The mid- to lower crustal signature is non-uniform across the profile. A ~ 35-km-wide, low-resistivity zone (C5: ~ 50–100 Ω·m) extends approximately from the CLLF to just south of the C3 feature. In contrast, the mid- to lower crust to the north (R2) is characterized by resistivity in the 500–1000 Ω·m range (Fig. 2D). Three subvertical features (C2-4) with lower resistivities of ≤ 50 Ω·m transect the higher resistivity upper crust (Fig. 2D). The C2 and C4 features coincide with the surface expressions of the Horne Creek and Porcupine-Destor faults, respectively. Although C3 does not correlate with any known major structure it connects, like C2, to C5 and thus may constrain the northern limit of the most VMS-endowed portion of the district. Depth slices at ~ 1000, 5500, 14,650, and 23,500 m reveal additional off-transect low-resistivity features (C6-7) transecting the upper crust, including C1 (Fig. 3a–d). Forming relatively discrete features at the surface, C1-3 and C6 amalgamate in the lower crust (≥ 20 km) in the broad low-resistivity C5 model feature that persist to depth. This is highlighted in the longitudinal section A-A’ along the Horne Creek fault showing the C2 feature localized to this structure and connection to C5 (Fig. 3f). Note the location of the Au-rich and Au-anomalous VMS deposits. A perspective view of the 3D MT model in the Noranda District shows low-resistivity isosurfaces define subvertical pipe-like features connected at depth to a broad subhorizontal low-resistivity zone, C5, itself potentially extending into the upper mantle (C8; Fig. 4). Calculating the shortest horizontal distance of each VMS deposit to the nearest 100 Ω·m contour at several depth slices in the resistivity model show that all VMS deposits remain within ~ 21 km to a depth of ~ 40 km (model cutoff) and on average within ~ 6 km (Supplementary Fig. S3; Supplementary Table S3). Notably, the two Au-rich VMS deposits are the only deposits that are consistently located within a 100 Ω·m contour at the ~ 1–2 km depth interval.

## Metal endowment

The crustal architecture of the Noranda District is characterized by a south-north asymmetry in geological and geophysical attributes and VMS endowment (Figs. 1, 2, 3, Supplementary S1, S2). The southern part of the district, located above C5 and between the CLLF and C3, contains ~ 104 Mt of the district’s ~ 130 Mt of VMS ore (15 of the 18 VMS deposits). This area has the greatest combined volume of synvolcanic TTG plutons and felsic volcanic rocks, and the highest density of major synvolcanic structures, all of which increase in relative abundance to the south and in particular in the southernmost Area 3 and 4. Gold-rich VMS deposits are restricted to the Powell and Horne blocks, and localized along the Horne Creek fault and C2 structure. The integration of the geophysical results corroborates the south-north asymmetry in surface geology and structure. The depth-extent of the Blake River assemblage is placed at the base of the high-resistivity upper crustal R1 zone (~ 14 km), an interpretation consistent with previous Lithoprobe seismic reflection profiles^26,27^ (Fig. 2E). The R1 zone does not distinguish between volcanic strata and synvolcanic intrusions, but the latter are well constrained by surface geology, drill holes, and mine data. However, intrusions such as the Flavrian, Powell, and Fabie produce homogenous low seismic reflectivity zones^28^, and thus, the 10–16 km depth extent of the low-reflectivity S1 zone south of C3 compared to 4–8 km to the north indicate the greater volume of synvolcanic intrusions localized to the former. This interpretation is supported by the density, surface geology, and previous work^29–31^ (Fig. 2, Supplementary Fig. S2).

The evolution of major structures bounding the district is debated, e.g., the CLLF has been interpreted as synvolcanic^15^, synorogenic^32^, and a linking of various structures of different origin^14^. Nonetheless, the density of synvolcanic structures and structural complexity are highest in the structural corridor between the CLLF and the C3 structure (Fig. 1; Supplementary Table S1), which contains the majority of VMS deposits (Fig. 2). Within this structural corridor an area of relatively low near-surface resistivity, C2, is localized to the Andesite, Horne Creek, and Beauchastel faults and continues in the subsurface as a near-vertical zone (~ 18 km deep) of discordant lower resistivity (Fig. 2D). The C2 faults are interpreted to be splays of the CLLF and collectively define the south margin of the district. The lower resistivity surface expression of C2 may be explained by an increased amount of sulfides and altered volcanic and intrusive rocks (Figs. 2, 3). The lower resistivity of C5 is consistent with grain boundary films of graphite, sulfide, or hydrous minerals within pathways where ascending magmas and fluid upflow were focused^21,33,34^. Feature C2 defines the pathway for magmas and metalliferous fluids located in deeper crustal to mantle sources (C5, C8; Figs. 2, 3, 4) to ascend to upper crustal sinks (throat of C2). Such a pattern is similar to that observed for the Olympic Dam mineral district^35^. This suggests that the three major synvolcanic faults comprising C2 define a focused transcrustral VMS hydrothermal upflow zone coincident with the largest and most Au-rich VMS deposits. The metal content in VMS deposits is thought to originate through leaching of crustal rocks by heated, modified seawater with the potential for periodic addition of magmatic fluids from sub-seafloor plutons emplaced within the roots of VMS hydrothermal systems^36–38^. As such, VMS formation and metal endowment are commonly thought of in the context of a shallow, sub-seafloor (~ < 5 km) convective, modified seawater dominated hydrothermal system. This conventional view has provided an explanation for the mild correlation observed between mid- to lower crustal conductors (100 Ω·m contours) and VMS deposits compared to a strong correlation between such conductors and orogenic gold deposits on a global scale, e.g., ~ 90% of orogenic gold deposits were found to be within 26 km of a 100 Ω·m contour at 20 km depth^9^. In the Noranda District, VMS and orogenic deposits are within 21.1 km and 22.8 km of a 100 Ω·m contour in the interval from ~ 1–40 km depth, respectively (Supplementary Table S3. The spatial association between VMS and orogenic Au deposits and low-resistivity features is consistent with both mineral systems sharing the same crustal infrastructure that resulted in endowment of the ~ 30 Ma older VMS deposits, e.g., as suggested for VMS and orogenic gold mineralization in the Bryah Rift Basin, Australia^10^. The location of the two Au-rich VMS deposits within a 100 Ω·m contour, highlights their proximity to where the low-resistivity pipe-like features come to surface (Supplementary Fig. S3). Thus, the integration presented here indicates that in the Noranda District, long-lived cross-stratal structural permeability was established early and maintained such that the subseafloor convective system is a near surface manifestation of a larger vertically extensive but areally restricted, deep crustal to mantle, magmatic-hydrothermal system (Figs. 2D, 3).

The relatively straight southern boundary to the C5 zone could correspond to a steeply north-dipping downward projection of the CLLF (Fig. 2C). The presence of the transcrustal structure is indicated on the density model at the abrupt change from greenstone to sedimentary rocks (Figs. 2D, S2).

The asymmetry of the Noranda District geology, crustal architecture, number of VMS deposits and the tenor of these deposits indicates that at the belt scale, the magmatic-hydrothermal centre for the Noranda volcanic complex was localized along a major, ancestral transcrustal structure and its splays, now represented by the CLLF, Andesite, Horne Creek, Beauschastel, and Hunter Creek faults (Fig. 2E). This structural corridor, continuously reactivated during volcanism, localized the large volumes of magma required to construct the Noranda volcanic complex. The anomalously high heat flow and deep crust-mantle cross-stratal permeability localized to the CLLF and its splays during construction of the Noranda volcanic complex likely resulted in the concentration, optimization, and sustainability of ore forming processes required to produce a world-class VMS district now expressed by the south-north asymmetry in the number, grade and tonnage of VMS deposits. Thus, similar to the formation of many giant porphyry Cu–Mo–Au deposits^39,40^, optimum circumstances rather than a unique mode of formation resulted in the extreme metal endowment of the Noranda District. This is highlighted by the less endowed part of the district, north of the C3 structure, which has fewer and smaller magmatic centers, lower fault density, fewer potential structural conduits of trans-lithospheric scale, and lacks deep crustal and mantle conductive zones.

The Horne Creek fault defines the southern limit of the magmatic centre for the Noranda volcanic complex (Figs. 1, 3). This is an optimal location for a magmatic contribution of metals to the VMS system that may explain the localization of Au-rich deposits to the Horne Creek fault throughout development of the Noranda volcanic centre. The younger (ca. 2698 Ma^12^) and world-class Au-rich, LaRonde Penna and Bousquet 2-Dumagami deposits in the Doyon-Bousquet-LaRonde (DBL) District, are located along the CLLF ~ 50 km east of the Noranda District^41^ (Fig. 1). Thus, two episodes of Au-rich VMS formation are localized to two separate volcanic complexes along the same transcrustal structure. These observations are consistent with the CLLF, or at least segments of it, originating as an extensional, synvolcanic transcrustal structure that localized metal endowed volcanic centers hosting the Noranda and DBL districts. Magmatism localized to this ancestral structure produced two major magmatic-hydrothermal centers and it is the proximity to this structure that resulted in the optimal conditions for high metal endowment, including Au-enrichment of VMS deposits. It is inferred that altered areas with a high density of early synvolcanic faults along this structure underwent preferential structural inversion and reactivation during later orogenesis that consequently localized orogenic Au mineralization (Fig. 1). As such, access to the deep core of a magmatic hydrothermal system might not in itself be sufficient to form syngenetic Au-rich VMS and subsequent orogenic Au deposits ca. 30 Ma later, but may also require successive involvement of melts and fluids sourced from an upper lithospheric mantle enriched in metals and Au^1,2,42,43^.

## Methods

Geological data were compiled and confirmed by field work along the ~ 80 km Metal Earth transect (Fig. 1). An estimate of VMS metal content, tonnage, Au grade, number of faults, and an areal analysis (ImageJ^44^) of the relative proportion of rock types were performed in each area (Figs. 1, 2A, Supplementary Fig. S1; Supplementary Table S1). A regional, deep seismic reflection profile covered a ~ 50 km segment of the transect centered on the Noranda District (Fig. 2B). Gravity data acquired along the transect were combined with existing Geological Survey of Canada regional compilations (http://gdrdap.agg.nrcan.gc.ca/) to generate a regional gravity grid. The VPmg algorithm^18,19^ was used to perform a smooth unconstrained 3-D gravity inversion providing a density cross-section (Fig. 2C) and a 3D isosurface model (Fig. S2). The geometry of the low density anomalies identified from density cross-section and corresponding isosurface model depends on the parameters used during inversion as well as sparsity of gravity data (Supplementary Fig. S4). New broadband (^~^10^–2^–10^3^ s bandwidth) MT data^20^ was combined with existing Lithoprobe data from the vicinity^22^, giving a total of 112 stations across the southern Abitibi region. Phase tensor dimensionality analysis^45^ (Supplementary Fig. S5) of the MT data shows the electrical structure (and hence geologic structure) to be 3D. The full-impedance tensor and vertical magnetic transfer functions were inverted using the HexMT algorithm^23,24^, resulting in a 3D resistivity model of the southern Abitibi, including the Noranda District. The inversion included full impedance tensor and vertical magnetic field data, as well as topography and solutions for the full distortion matrix. A final nRMS of 2.19 was reached with misfit distribution evenly among the stations. Sensitivity tests were performed individually on significant features by replacing the resistivities of the corresponding region with resistivities representative of the relevant depths^20^. A slightly meandering slice through the Noranda District 3D resistivity volume follows the MT station distribution in the Noranda District and the seismic transect (Fig. 2D). Further detail on the methodology for the geophysical surveys are provided in Supplementary Appendix DR2.

## Supplementary Information

Supplementary Information.

## Data Availability

Original geophysical datasets generated during the current study are available from the Mineral Exploration Research Centre (MERC) repository: Jørgensen et al. 2022.
