# Supplementary material for: The implications of crustal architecture and transcrustal upflow zones on the metal endowment of a world-class mineral district
Source: Sci Rep. 2022 Aug 29;12:14710. doi: 10.1038/s41598-022-18836-y (PMC9424197; doi:10.1038/s41598-022-18836-y)
Supplement: Supplementary file 1 — Supplementary Information. [file 41598_2022_18836_MOESM1_ESM.pdf]

Appendix DR1. EXPANDED GEOLOGICAL SETTING

The Noranda District is a mature mining district located in Quebec, Canada (Fig. 1; Gibson and Galley, 2007). The VMS hosting Noranda volcanic centre belongs to the 2704-2695 Ma Blake River episode, the youngest volcanic episode of the Abitibi subprovince, southeastern Superior Province (Fig. 1; Thurston et al., 2008; McNicoll et al., 2014). The Blake River episode at Noranda comprise a bimodal, tholeiitic to transitional basalt-dominated, central volcanic complex with subordinate transitional to calc-alkaline, FIII rhyolites and lesser dacites and rhyolites that is localized along the Larder Lake-Cadillac fault zone to the south (Lesher et al., 1986; Hart et al., 2004). This fault dissects the Abitibi subprovince, and juxtaposes the Noranda volcanic centre against the Pontiac subprovince, dominantly comprised of ~2682 Ma metawackes that unconformably underlies sedimentary rocks of the Timiskaming assemblage (~2672-2665 Ma; Fig. 1) (e.g., Ludden et al. 1982; Dimroth et al. 1982; Gélinas et al. 1984; Gibson and Watkinson, 1990). To the north the Porcupine-Destor fault juxtaposes the Blake River group against the 2723-2720 Ma Stoughton-Roquemaure volcanic episode (Thurston et al., 2008; Jørgensen et al., 2018). Both faults are auriferous transcrustal structures (100s of km long) with a prolonged and complex kinematic history (e.g., Dimroth, 1982; Bleeker, 2015; Lafrance, 2015; Poulsen, 2017; Bedeaux et al., 2017). The main phase of post-Timiskaming (~2672-2665 Ma) north-south shortening produced the dominant east-west structural grain observed in rocks proximal to the faults (e.g., Gibson and Galley, 2007; Bedeaux et al., 2017). In general, deformation decreases in the Blake River assemblage and Noranda volcanic centre with increasing distance from the transcrustal bounding faults (e.g., Gibson and Galley, 2007). Major subvertical faults dissect strata of the Noranda volcanic centre, particularly in the southern part of the district where they define distinct structural blocks (Fig. 1; Spence, 1976; Péloquin et al., 1990). These include the Andesite, Horne Creek, Powell, Beauchastel, Hunter Creek and Baie Fabie faults (Fig. 1), all of which are interpreted to be reactivated synvolcanic faults based on abrupt facies changes, restriction/truncation of some units, localized VMS alteration, the occurrence of synvolcanic rhyolite and basalt dikes that occupy some structures, and spatial association (localization) of synvolcanic subvolcanic intrusions (Spence and de Rosen Spence, 1975; de Rosen-Spence, 1976; Dimroth, 1982, Setterfield et al.1987; 1995; Gibson, 1990; Gibson & Watkinson, 1990; Kerr and Gibson, 1993;

Péloquin et al., 1990; Moore et al., 2016). The large, sill-like, Flavrian and Powell, and smaller Fabie subvolcanic tonalite-trondhjemite-granodiorite plutons (ca. 2701 Ma) define magmatic centres within the Noranda volcanic complex that are localized, terminate and offset along these faults (Goldie, 1979; Galley, 2003; McNicoll et al., 2014). These intrusions are integral components of VMS ores systems and are interpreted to be important heat sources necessary to localize and drive high temperature convective modified seawater hydrothermal systems responsible for VMS formation and/or a direct source of metals to the VMS ore system (Gibson and Kerr, 1993; Galley, 2003; Franklin et al., 2005). The inferred geodynamic setting for the Noranda volcanic centre is an immature arc-back-arc (e.g., Dimroth, 1982; Yang and Scott, 2003; Mercier-Langevin et al., 2007), where the FIII felsic rocks are interpreted to be the products of shallow crustal melting of tholeiitic basalts (Hart et al., 2004).

The distribution of VMS deposits within the Noranda volcanic centre is not uniform (Fig. 1). The smaller Magusi and Fabie deposits (<5 Mt; <2 g/t Au; Gibson and Galley, 2007) are the only deposits found in the Hunter block bound by the Porcupine-Destor and Hunter Creek faults, and no economic VMS occurrence have been found north of the Baie Fabie fault (Fig. 1), which may define the northern limit of the Noranda VMS district. The majority of deposits occur in the Flavrian block, bound by the Hunter Creek and Beauchastel faults, and are all <10 Mt and contain ≤2 g/t Au (Fig. 1; Gibson and Galley, 2007). The Powell block, bound by the Beauchastel and Horne Creek faults, hosts the Au-rich Quemont (16.65 Mt; 5.50 g/t Au) and two small deposits (<1 Mt) with elevated Au-grades of 2.40 g/t (Delbridge) and 4.10 g/t (Deldona) (Fig. 1; Gibson and Galley, 2007). The Horne Creek and Andesite faults bound the Horne block that hosts Au-rich Horne deposit (54.3 Mt; 6.10 g/t Au) that is recognized as the world's largest Au-rich VMS deposit and categorizes as world-class deposit (> 100 t Au: Singer, 1995; Fig. 1). Thus, the four VMS deposits with the highest Au-grades in the Noranda District lie at different stratigraphic intervals along the Horne Creek fault, a splay of the nearby Larder Lake-Cadillac fault zone.

Notably, none of the VMS deposits north of the Beauchastel fault grade ≥2.00 g/t Au. The Rouyn-Pelletier block sits between the Andesite and Larder Lake-Cadillac faults and hosts no known VMS deposits. The Bouchard-Hebert deposit, although included in the Noranda District, is located east of the D'Alembert fault, a structure that is continuous with the Baie Fabie fault and defines the eastern limit to most structural blocks of the Noranda Volcanic centre (Fig. 1). The Bouchard-Hebert deposit occurs within the youngest ca 2695 Ma volcanic succession of the Blake River episode and may comprise a discrete, separate and localized volcanic centre (McNicoll et al., 2014; Pearson and Daigneault, 2009; Mueller et al., 2012). The ~20 orogenic Au deposits and occurrences in the Noranda District are mainly hosted along the Larder Lake-Cadillac fault zone, but are also associated with subsidiary structures within the Noranda volcanic centre and the Flavrian and Powell synvolcanic plutons (Fig. 1; e.g., Carrier et al., 2000; Poulsen, 2017). The intrusion-hosted, synvolcanic Cu-Mo ±Au ±Ag mineralization is hosted by the Flavrian and Powell plutons (Fig. 1; e.g., Goldie et al., 1979).

Many high-precision U-Pb ages available in the Noranda volcanic centre demonstrate that VMS mineralization formed during two stages of its ca. 2704-2698 volcanic evolution (McNicoll et al., 2014). The ca. 2702 Ma Au-rich Horne and Quemont deposits are among the oldest VMS deposits in the Noranda District, whereas VMS deposits in the Flavrian block are hosted by ca. 2702-2701 Ma and ca. 2698 Ma rocks (McNicoll et al., 2014). Furthermore, available ages now show that the oldest volcanic rocks occur in the outer exposed areas of the Blake River assemblage and margins of the Noranda volcanic centre, whereas the youngest rocks are dominantly confined to the Flavrian block between the Flavrian pluton and the D'Alembert fault (McNicoll et al., 2014). Although basalts to basaltic andesites are abundant throughout the bimodal mafic volcanic succession hosting the VMS deposits of the Noranda District, the ratio of

mafic to felsic rocks is noticeably different from north to south (Fig. 1). Only one significant felsic center occurs north of the Baie Fabie shear zone, with an increase in felsic volcanic rocks as the Hunter Creek fault is approached from the north, and the highest proportion of felsic rocks are located to the south in the Powell and Horne structural blocks. Similarly, the area occupied by subvolcanic intrusions is much greater in the southern Flavrian and Powell blocks (Flavrian and Powell plutons respectively) compared to the Hunter block (Fabie pluton; Fig. 1). The density of synvolcanic faults is higher in the south relative to areas north of the Hunter Creek fault (Fig. 2; Spence and de Rosen Spence, 1975; Dimroth, 1982, Gibson & Watkinson, 1990; Kerr and Gibson, 1993; Péloquin et al., 1990; Moore et al., 2016). South of and including the Hunter Creek fault, all other major re-activated synvolcanic structures (Beauchastel, Powell, Horne Creek, and the Andesite) are splays of the Larder Lake-Cadillac fault (Fig. 1).

REFERENCES CITED

Bedeaux, P., Pilote, P., Daigneault, R., and Rafini, S., 2017, Synthesis of the structural evolution and associated gold mineralization of the Cadillac fault, Abitibi, Canada: Ore Geology Reviews, v. 82, p. 49–69, doi: 10.1016/j.oregeorev.2016.11.029.

Bleeker, W., 2015, Synorogenic gold mineralization in granite-greenstone terranes: The deep connection between extension, major faults, synorogenic clastic basins, magmatism, thrust inversion, and long-term preservation: Geological Survey of Canada Open File 7852, p. 25–47.

Carriere, C., Jébrak, M., Angelier, J., and Holyland, P., 2000, The Silidor Deposit, Rouyn-Noranda District, Abitibi Belt: Geology, Structural Evolution, and Paleostress Modeling of

an Au Quartz Vein-Type Deposit in an Archean Trondhjemite: Economic Geology, v. 95, p. 1049-1065, doi: 10.2113/gsecongeo.95.5.1049.

de Rosen-Spence, A.F., 1976, Stratigraphy, development and petrogenesis of the central Noranda volcanic pile, Noranda, Quebec [Ph.D. thesis]: Toronto, Ontairo, University of Toronto, 166 p.

Dimroth, E., Imreh. L., Rocheleau, M., and Goulet, N., 1982, Evolution of the south central part of the Archean Abitibi Belt, Quebec. Part 1: Stratigraphy and paleogeographic model: Canadian Journal of Earth Sciences, v. 19, p. 1729-1758, doi: 10.1139/e82-154.

Franklin, J.M., Gibson, H.L., Jonasson, I.R., and Galley, A.G., 2005, *in* Hedenquist, J.W., Thompson, J.F.H., Goldfarb., R.J., and Richards, J.P., eds., Economic Geology 100th Anniversary Volume: Littleton, Colorado, p. 523-560, doi: 10.5382/AV100.17.

Galley, A.G., 2003, Composite synvolcanic intrusions associated with Precambrian VMS-related hydrothermal systems: Mineralium Deposita, v. 38, p. 443-473, doi: 10.1007/s00126-002-0300-9.

Gélinas, L., Trudel, P., and Hubert, C., 1984, Chemostratigraphic subdivision of the Blake River Group, Rouyn-Noranda area, Abitibi, Quebec: Canadian Journal of Earth Sciences, v. 21, p. 220-231, doi: 10.1139/e84-023.

Gibson, H.L., 1990, The mine sequence of the Central Noranda Volcanic Complex: Geology, alteration, massive sulphide deposits and volcanological reconstruction [Ph.D. thesis]: Ottawa, Canada, Carleton University, 715 p.

Gibson, H.L., Watkinson, D.H., 1990, Volcanogenic massive sulphide deposits of the Noranda cauldron and shield volcano, Quebec, *in* Rive, M., Verpaelst, P., Gagnon, Y., Lulin, J.M., Riverin, G., and Simard, A., eds., The northwestern Quebec polymetallic belt: A summary

of 60 years of mining exploration: Montreal, Quebec, The Canadian Institute of Mining and Metallurgy Special Volume 43, p. 119–132.

Gibson, H.L., and Galley, A.G., 2007, Volcanogenic massive sulphide deposits of the Archean, Noranda District, Quebec, *in* Goodfellow, W.D., ed., Mineral deposits of Canada: a synthesis of major deposit-types, district metallogeny, the evolution of geological provinces, and exploration methods: St. John, Newfoundland and Labrador, Canada, Special Publication 5, Mineral Deposits Division, Geological Association of Canada, p. 533-552.

Goldie, R.J., 1976, A geological investigation of the Flavrian and Powell plutons and their relationships to other rocks and structures of the Noranda area, the Flavrian and Powell plutons, Noranda area, Quebec [Ph.D. thesis]: Kingston, Canada, Queen's University, 354 p.

Hart, T., Gibson, H.L. and Lesher, C.M., 2004, Trace element geochemistry and petrogenesis of felsic volcanic rocks associated with volcanogenic Cu-Zn-Pb massive sulphide deposits: Economic Geology, v. 99, p. 1003-1013, doi: 10.2113/gsecongeo.99.5.1003.

Jørgensen, T.R.C., Gibson, H.L., Hamilton, M.A., 2018, U-Pb zircon geochronology and geochemistry of volcanic rocks in the Deguisier Formation, Abitibi Greenstone Belt, Quebec: implications for gold and VMS mineralization: Society of Economic Geology 2018 – Metals, Minerals and Society, Abstract P030.

Kerr, D.J., and Gibson, H.L., 1993, A comparison of the Horne volcanogenic massive sulphide deposit and intracauldron deposits of the Mine Sequence, Noranda, Quebec: Economic Geology, v. 88, p. 1419-1442, doi: 10.2113/gsecongeo.88.6.1419.

Lafrance, B., 2015, Geology of the orogenic Cheminis gold deposit along the Larder Lake-Cadillac deformation zone, Ontario: Canadian Journal of Earth Sciences, v. 52, p. 1093–1108, doi: 10.1139/cjes-2015-0067.

Lesher, C.M., Goodwin, A.M., Campbell, I.H., and Gorton, M.P., 1986, Trace-element geochemistry of ore-associated and barren, felsic metavolcanic rocks in the Superior Province, Canada: Canadian Journal of Earth Sciences, v. 23, p. 222-237, doi: 10.1139/e87-143.

Ludden, J.N., Gélinas, L., and Trudel, P., 1982, Archean metavolcanics from the Rouyn-Noranda District, Abitibi Greenstone Belt, Quebec. 2. Mobility of trace elements and petrogenetic constraints: Canadian Journal of Earth Sciences, v. 19, p. 2276-2287, doi: 10.1139/e82-200.

McNicoll, V., Goutier, J., Dubé, B., Mercier-Langevin, P., Ross, P-S., Dion, C., Monecke, T., Legault, M., Percival, J., and Gibson, H.L., 2014, U-Pb geochronology of the Blake River Group, Abitibi Greenstone Belt, Quebec, and implications for base metal exploration: Economic Geology, v. 109, p, 27–59, doi: 10.2113/econgeo.109.1.27.

Mercier-Langevin, P., Dubé B., Hannington M.D., Richer-Laflèche M., and Gosselin G., 2007, The LaRonde Penna Au-rich volcanogenic massive sulfide deposit, Abitibi Greenstone Belt. Quebec: Part II. Lithogeochemistry and paleotectonic setting: Economic Geology, v. 102, p. 611–631, doi: 10.2113/gsecongeo.102.4.611.

Moore, L.N., Daigneault, R., Aird, H.M., Banerjee, N.R., and Mueller W.U., 2016, Reconstruction and evolution of Archean intracaldera facies: the Rouyn–Pelletier Caldera Complex of the Blake River Group, Abitibi greenstone belt, Canada: Canadian Journal of Earth Sciences, v. 53, p. 355-377, doi: 10.1139/cjes-2015-0029.

Mueller, W.U., Friedman, R., Daigneault, R., Moore, L., and Mortensen, J., 2012, Timing and characteristics of the Archean subaqueous Blake River Megacaldera Complex, Abitibi greenstone belt, Canada: Precambrian Research, v. 214-215, p. 1-27, doi: 10.1016/j.precamres.2012.02.003.

Pearson, V., and Daigneault, R., 2009, An Archean megacaldera complex: the Blake River Group, Abitibi greenstone belt: Precambrian Research, v. 168, p. 66−82, doi: 10.1016/j.precamres.2008.03.009.

Péloquin, A.S., Potvin, R., Paradis, S., Laflèche, M.R., Verpaelst, P., and Gibson, H.L., 1990, The Blake River Group, Rouyn-Noranda area, Québec: A stratigraphic synthesis, *in* Rive, M., Verpaelst, P., Gagnon, Y., Lulin, J.M., Riverin, G., and Simard, A., eds., The northwestern Québec polymetallic belt: A summary of 60 years of mining exploration: Montreal, Quebec, The Canadian Institute of Mining and Metallurgy Special Volume 43, p. 107–118.

Poulsen, K.H., 2017, The Larder Lake-Cadillac Break and its gold districts: *in* Monecke, T., Mercier-Langevin, P., and Dubé, B., eds., Archean base and precious metal deposits, southern Abitibi Greenstone Belt: Littleton, Colorado, Reviews in Economic Geology 19, p. 133–167, doi: 10.5382/Rev.19.05.

Setterfield, T., 1987, Massive and brecciated dikes in the McDougall and Despina faults, Noranda, Quebec, Canada: Journal of Volcanology and Geothermal Research, v. 31, p. 87–97, doi: 10.1016/0377-0273(87)90007-2.

Setterfield, T.N., Hodder, R.W., Gibson, H.L., and Watkins, J.J., 1995, The McDougall-Despina fault set, Noranda, Quebec: evidence for fault-controlled volcanism and hydrothermal fluid flow: Exploration and Mining Geology, v. 4, p. 381-393.

Singer, D.A., 1995, World class base and precious metal deposits: A quantitative analysis: Economic Geology, v. 90, p. 88–104, doi: 10.2113/gsecongeo.90.1.88.

Spence, A.F., 1976, Stratigraphy, development and petrogenesis of the central Noranda volcanic pile, Noranda, Quebec [Ph.D. thesis]: Toronto, Ontario, Canada, University of Toronto, 166 p.

Spence, C.D., and de Rosen-Spence, A.F., 1975, The place of sulfide mineralization in the volcanic sequence at Noranda, Quebec: Economic Geology, v. 70, p. 90–101, doi: 10.2113/gsecongeo.70.1.90.

Thurston, P.C., Ayer, J.A., Goutier, J., and Hamilton M.A., 2008, Depositional gaps in Abitibi Greenstone Belt stratigraphy: a key to exploration for syngenetic mineralization: Economic Geology, v. 103, p. 1097–1134, doi: 10.2113/gsecongeo.103.6.1097.

Yang, K., and Scott, S.D., 2003, Geochemical relationships of felsic magmas to ore metals in massive sulfide deposits of the Bathurst mining camp, Iberian Pyrite Belt, Hokuroku district, and the Abitibi Belt, *in* Goodfellow, W.D., McCutcheon, S.R., and Peter, J.M., eds., Massive sulfide deposits of the Bathurst Mining Camp, New Brunswick, and Northern Maine: Littleton, Colorado, Economic Geology Monograph 11, p. 457–478.

Supplementary Appendix DR2. GEOPHYSICAL METHODS

A regional, deep seismic reflection profile was designed to image the structural architecture of the crust and covered a ~50 km transect centered on the Noranda District (Fig. 2b). Cheraghi et al. (2018) and Naghizadeh et al. (2019) presents the specifications of the survey and the processing performed to produce the migrated profile in Figure 2. In summary, an array of four vibrator trucks producing a linear upsweep of 2–96 Hz was repeated four times at each nominal source location. Surveying used 50 m source (4 sweeps) and 25 m receiver intervals. Acquisition parameters were a 12 or 16 s recording length, 2 ms sample rate, 15 km— 0—15 km spread size, 5 Hz geophones (single), and AHV-IV 364 Commander vibrator trucks (Naghizadeh et al., 2019). The seismic data processing stream included trace kills and reversals, minimum phase conversion, ensemble balance and amplitude recovery, surface consistent scaling, linear and erratic noise attenuation, surface-consistent deconvolution, anomalous frequency suppression, refraction statics, velocity analyses, surface consistent residual statics, and post- and pre-stack time migrations (Naghizadeh et al., 2019). There is some overlap with seismic transects (line 21 and 21-1) conducted across the Superior craton ~30 years ago as part of the Lithoprobe program (Calvert & Ludden, 1999; Percival & West, 1994; White et al., 2003). Overlaps between the two surveys show better resolution of the upper crust along the Metal Earth seismic profiles (Naghizadeh et al., 2019).

Gravity data were acquired from transect stations with an average spacing of 300 m and side road stations up to 1 km from the transect (Maleki et al., 2020). The data were combined with existing Geological Survey of Canada regional compilation (http://gdrdap.agg.nrcan.gc.ca/) and are corrected for changes in latitude, elevation using software *Oasis Montaj*® (https://www.seequent.com/products-solutions/geosoft-oasis-montaj/). Terrain corrections were applied assuming background density of 2.67 g/cm^3. The VPmg software Version 9.0 (Fullagar and Pears, 2007; Fullagar, 2013) linked to Mira Geoscience GOCAD mining suite was used to perform a smooth unconstrained 3D gravity inversion. The initial 3D density model was constructed within a volume with dimensions of ~(84 km x 104 km x 15 km) and a 450 m cell size. Topographic information (SRTM 90m) used for this inversion was

downloaded from Geosoft public DAP server (http://dap.geosoft.com). The inversion started with initial density contrast value of 0.01 g/cm^3 for all the cells in the subsurface model and allowed VPmg to numerically change the property of each cell to fit the observed data. The lower and upper density contrast values are fixed during inversion i.e. 0 and 1. The final unconstrained inversion of gravity data, after 78 iterations, returned the RMS misfit of 1 mGal from an initial value of 10.68 mGal. The density cross-section (Fig. 2c) was extracted along the transect from the final inverted 3D density model. A 3D isosurface model (Fig. S2) was generated using the density contrast value of 0.07 g/cm^3. The density cross-section delineates several low-density anomalies (G1-G6) and corresponding 3-D isosurface model provides geometry of low-dense features. The smooth unconstrained potential-field inversions suffer with problem of non-uniqueness (Fullagar and Pears, 2007; Li and Oldenberg, 1998) and hence should be combined with other geophysical methods for a plausible geological interpretation.

Metal Earth contracted the collection of 750 new MT measurements through Complete Magnetotelluric Solutions and Moombarriga Geoscience, comprised of a combination of broadband- and audio- MT soundings using the Phoenix Geophysics V5-2000 MT system. When the newly collected MT data set is combined with the existing MT data sets there are upwards of 1100 MT soundings in the area of interest. A total of 13 'regional' north-south MT transects ranging in length from 60-150 km were acquired (Hill et al. 2021; Roots et al., 2022), which when combined with existing data sets such as *Lithoprobe* (Jones et al., 2014) provide an areal coverage of > 2.5×105 km^2. Nested within these 'regional' transects are more densely sampled high-resolution segments up to ~10 km in length to provide improved discrimination of lateral upper crustal structure in zones of localized strain that are commonly coincident with mineralised domains. Broadband-MT data spans the frequency range of ~320-0.001 Hz, depths of up to ~75 km given favourable survey conditions (Chave and Jones, 2012). While the audio-MT data spans the frequency range, as the name implies, from the audio band in which sound waves are detectable to the human ear (~10000-1 Hz), a depth range of ~0.5-3 km can be imaged given the resistive near surface (Chave and Jones, 2012). MT soundings were collected primarily along profiles; however, a

number of soundings were collected offset from the main profile trace. These stations, combined with the pre-existing data, comprise a ‘swath’ or ‘corridor’ transect, giving improved 3D control. The regional transects have a station spacing of ~5 km and are comprised solely of broadband MT soundings with observation recordings of 24-48 hours. The high-resolution segments within the regional transects contain alternating broadband- and audio-MT soundings with a station spacing of ~330 m and recording times of ~24 hours for broadband MT soundings and ~4 hours for audio-MT soundings. All time series data collected within Metal Earth were processed to obtain estimates of the frequency domain transfer functions Z and K using robust remote reference processing (Jones et al., 1989) from a distal contemporaneously recording site.

Phase tensor dimensionality (Caldwell et al., 2004; Bibby et al., 2005; Booker 2014) analysis (Fig. S3) of the MT data show the electrical structure (and hence geologic structure) to be 3D with β (a rotational invariant indicative of dimensionality) values greater than the ± 3° threshold for 1D & 2D structure (Caldwell et al., 2004; Bibby et al., 2005; Booker 2014), necessitating a 3D approach to analysis and inverse modelling. All of the components used in the inverse computations (Z, and K) contain information that allows identification of structure away from the main transect trace. This off-transect 3D structure is required to honour the 3D nature of the observed data identified by phase tensor analysis. Longer wavelengths probe deeper and farther from the observation location; however, the resolution correspondingly lessens with both depth and distance (Chave and Jones, 2012). Inverse modelling of all of the ‘swath’ or ‘corridor’ transects in the Upper Abitibi region supplemented by extant Lithoprobe data (Jones et al., 2014) allows treatment of the meandering profiles including off-transect soundings. HexMT (Kordy et al., 2016a,b; Wannamaker et al., 2017) was used to compute the inverse models presented. Inversion was conducted on 112 sites with 18 logarithmically spaced periods between 144-0.0011 Hz and error floors on the real and imaginary parts of the complex impedance elements Zij applied of max (assigned Zij error, 5%(|Zxy-Zyx|/2)) at each frequency. The starting and apriori model for inversions was a 600 Ωm half-space mesh consisting of 207x237x59 nodes and a nominal horizontal cell size of ~1.2 km

with areas of local refinement. The preferred best fit model with static shift solution had an nRMS of 2.18. The displayed sections follow the meandering nature of the measured Rouyn-Noranda MT soundings. The 3D inversion closely reproduces all modelled components of the observed data.

The HexMT algorithm (Kordy et al., 2016a,b; Wannamaker et al., 2017), exploits deformable hexahedral finite elements (FE) to precisely incorporate topography. It uses direct solvers parallelized on symmetric multiprocessor (multi-core) workstations with large RAM for both the forward solution, parameter Jacobians, and the parameter model update. First-order edge elements are used to represent the secondary electric field (E), yielding accuracy O(h) for E and its curl (magnetic field). A divergence correction for the E-field is applied using the single-step Hodge decomposition. The system matrix factorization and source vector solutions are computed directly using the MKL PARDISO library. The factorized matrix is used to calculate the forward response as well as the Jacobians of electromagnetic field and MT responses using the reciprocity theorem. By exploiting the data-space approach, the computational cost of the model update is greatly reduced in both time and computer memory compared to that of the model-space forward simulation. In order to regularize the inversion using the L2 norm of the gradient, we factor the matrix related to the regularization term and apply its inverse to the Jacobian, which is also performed using the MKL PARDISO library. For dense matrix multiplication and factorization related to the Gauss–Newton model update, we use the PLASMA library. The libraries show good scalability across processor cores.

REFERENCES CITED

Calvert, A. J., and Ludden, J. N. ,1999, Archean continental assembly in the southeastern Superior Province of Canada: Tectonics, v. 18, p. 412–429.

Cheraghi, S., Naghizadeh, M., Snyder, D., & Mathieu, L. ,2018, Crustal-scale seismic investigation in Chibougamau, Quebec, Canada. In Near Surface Geoscience 2018 (EAGE),

Workshop: worldwide Mineral Exploration Challenges and Cost-effective Geophysical Methods, Porto, Portugal. Abstract.

Cheraghi, S., Naghizadeh, M., Snyder, D., Haugaard, R., and Gemmell, T., 2019, High resolution seismic imaging of crooked two dimensional profiles in greenstone belts of the Canadian shield: results from the Swayze area, Ontario, Canada. Geophysical Prospecting.

Fullagar, P. K., 2013. VPmg User Documentation, Version 7.1. (Fullagar Geophysics Pty Ltd, Report FGR01F-4).

Fullagar, P. K., and Pears, G. A., 2007, Toward geologically realistic inversion, Proceedings of Exploration 07: Fifth Decennial International Conference on Mineral Exploration.

Hill, G. J. et al., 2021, On Archean craton growth and stabilisation: Insights from lithospheric resistivity structure of the Superior Province: Earth Planet. Sci. Lett., v. 562, 116853.

Jones A.G., Ledo, J., Ferguson, I.J., Craven, J.A., Unsworth, M.J., Chouteau, M., and Spratt, J.E., 2014, The electrical resistivity of Canada's lithosphere and correlation with other parameters: contributions from Lithoprobe and other programmes: Canadian Journal of Earth Sciences, v. 51, p. 573-617, doi: 10.1139/cjes-2013-0151.

Kordy, M., Wannamaker, P., Maris, V., Cherkaev, E., and Hill, G., 2016a, 3-D magnetotelluric inversion including topography using deformed hexahedral edge finite elements and direct solvers parallelized on SMP computers – Part I: forward problem and parameter Jacobians: Geophysical Journal International, v. 204, p. 74–93, doi: 10.1093/gji/ggv410.

Kordy, M., Wannamaker, P., Maris, V., Cherkaev, E., and Hill, G., 2016b, 3-dimensional magnetotelluric inversion including topography using deformed hexahedral edge finite elements and direct solvers parallelized on symmetric multiprocessor computers – Part II:

direct data-space inverse solution: Geophysical Journal International, v. 204, p. 94–110, doi: 10.1093/gji/ggv411.

Li, Y., Oldenberg, D.W., 1998, 3-D inversion of gravity data: Geophysics 63 (1), 109-119.

Maleki, A., Smith, R. S., Eshaghi, E., Mathieu, L., Snyder, D., and Naghizadeh, M., 2020, Potential-field modelling of the prospective Chibougamau area (NE Abitibi subprovince, Quebec), using geological, geophysical and petrophysical constraints: Canadian Journal of Earth Sciences, *in press*, doi: 10.1139/cjes-2019-0221.

Naghizadeh, M., Snyder, D., Cheraghi, S., Foster, S., Cilensek, S., Floreani, E., and Mackie, J., 2019, Acquisition and Processing of Wider Bandwidth Seismic Data in Crystalline Crust: Progress with the Metal Earth Project: Minerals, 9, 145.

Percival, J. A., and West, G. F. ,1994, The Kapuskasing uplift: a geological and geophysical synthesis: Canadian Journal of Earth Sciences, v. 31, p. 1256–1286.

Roots, E. et al., 2022, Magmatic, hydrothermal and ore element transfer processes of the southeastern Archean Superior Province implied from electrical resistivity structure: Gondwana Research, v. 105, p. 84-95.

White, D. J., Musacchio, G., Helmstaedt, H. H., Harrap, R. M., Thurston, P. C., Van der Velden, A., and Hall, K. ,2003, Images of a lower-crustal oceanic slab: Direct evidence for tectonic accretion in the Archean western Superior province: Geology, v. 31, p. 997–1000.

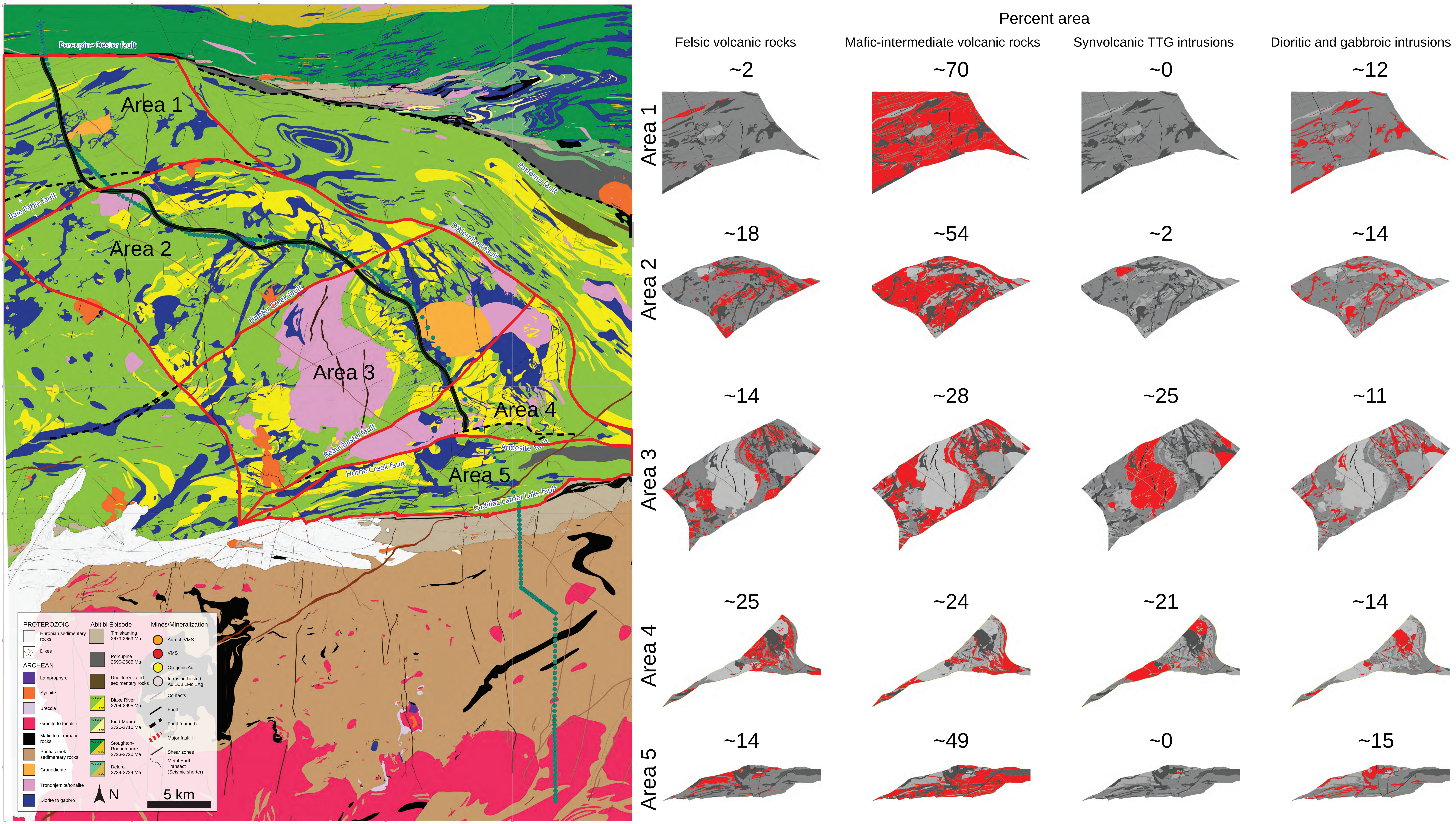

Author name: Taus R. C. Jørgensen et al.

Figure number: S1

Manuscript title: The implications of crustal architecture and transcrustal upflow zones on the metal endowment of a world-class mineral district

Caption: Figure S1. Surface area analyzes performed in five different areas along the Metal Earth Transect to compare relative areas of felsic volcanic rocks, mafic-intermediate volcanic rocks, synvolcanic trondhjemite-tonalite-granodiorite (TTG) intrusions, and diorite-gabbro intrusions. Area 1 and 2 are the Hunter block devided into a northern and southern portion at the Baie Fabie fault. Area 3 is equivalent to the Flavrian block, Area 4 is the Powell and Horne blocks combined, and Area 5 is the Rouyn-Pelletier block. The Figure was compiled in Adobe Illustrator (≥ v. CC2018 22.0.0) with the geological map generated using open data downloaded from Système d'information géominière of Québec (SIGÉOM; https://sigeom.mines.gouv.qc.ca/signet/classes/I1102_aLaCarte?l=a#) and insets of percent area generated in open source application ImageJ (https://imagej.nih.gov/ij/).

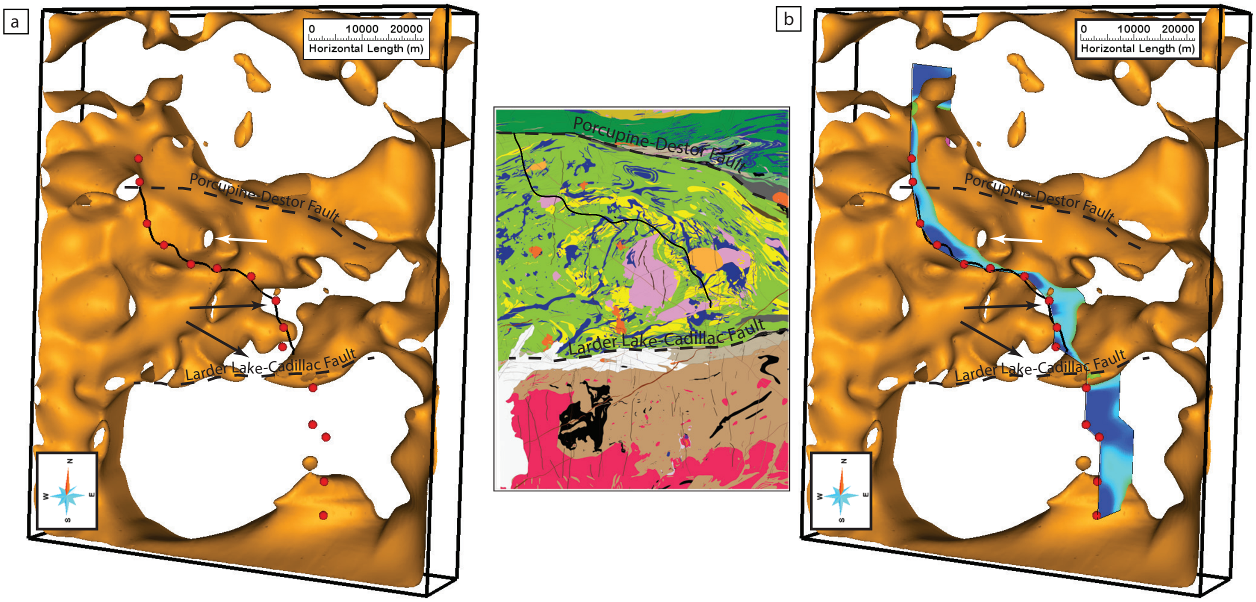

Author name: Taus R. C. Jørgensen et al.

Figure number: S2

Manuscript title: The implications of crustal architecture and transcrustal upflow zones on the metal endowment of a world-class mineral district

Caption: Figure S2. a) 3-D isosurface of high density (>0.07 density value) region with MT survey locations and seismic survey line overlaid. b) 3-D isosurface of high density (>0.07 density value) region with cross section. Black and white arrows indicate low density regions along the Rouyn-Noranda transect between the major faults. The inset is Figure 1 for reference. The Emerson Paradigm software SKUA-GOCAD-17 (https://www.pdgm.com/products/skua-gocad) was used for 3D visualization.

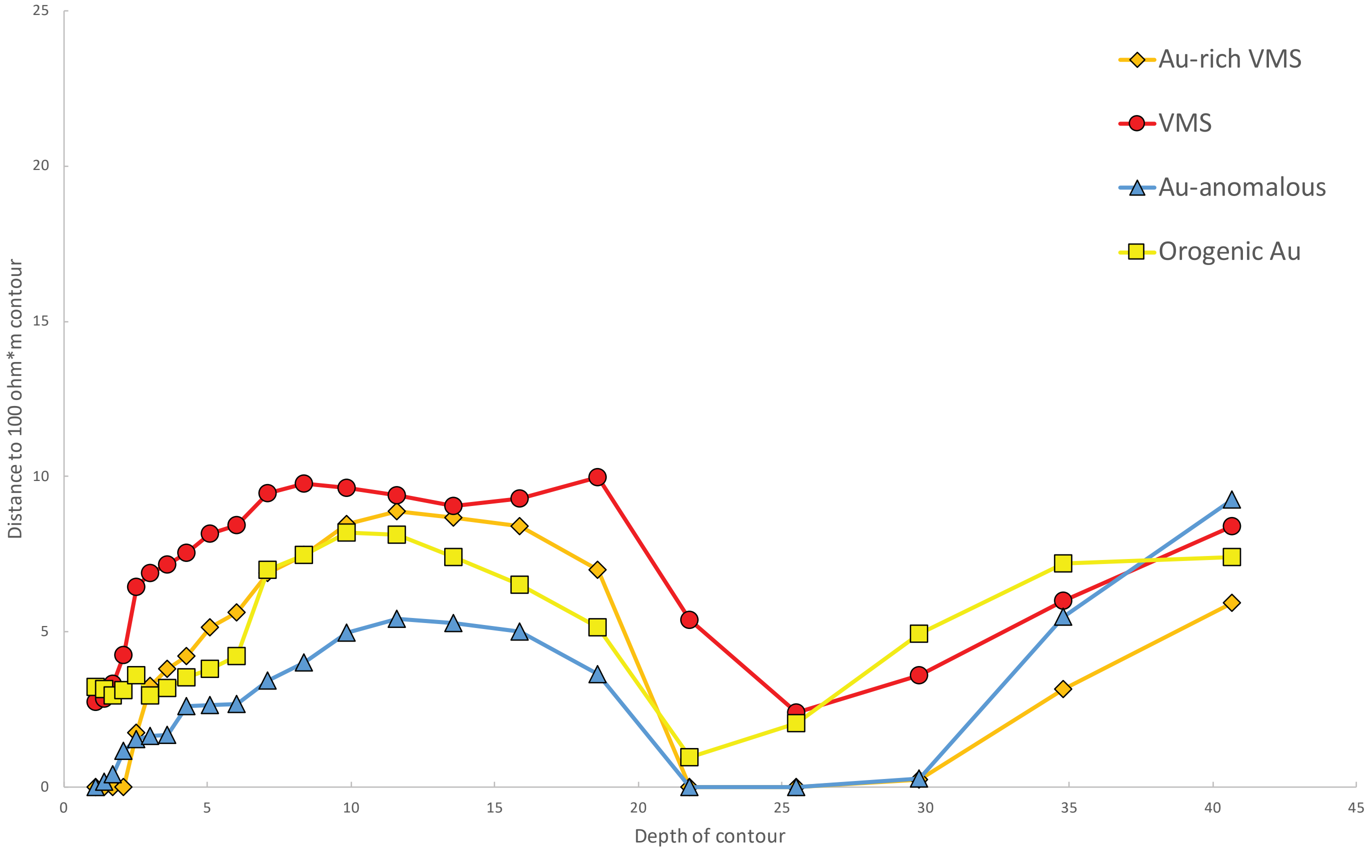

Author name: Taus R. C. Jørgensen et al.

Figure number: S3

Manuscript title: The implications of crustal architecture and transcrustal upflow zones on the metal endowment of a world-class mineral district

Caption: Figure S3. Diagram showing the average distance to a 100 Ω·m contour versus the depth of the contour for Au-rich VMS (n=2), Au-anomalous VMS (n=2), conventional VMS (n=14), and orogenic gold (n=14) deposits. Note that deposits falling within a 100 Ω·m contour have a distance of 0 m instead of giving a negative distance that might skew the data.

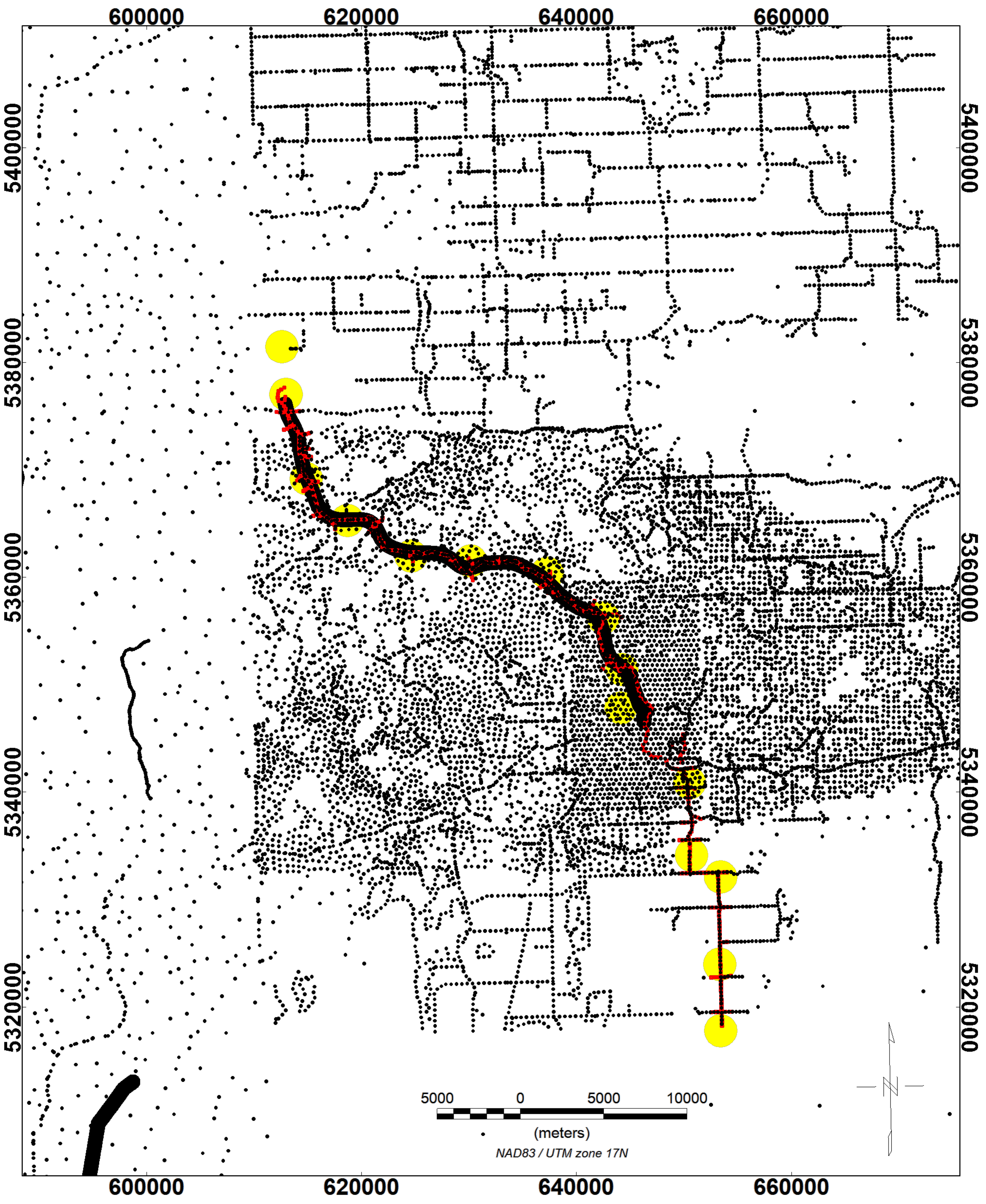

Author name: Taus R. C. Jørgensen et al.

Figure number: S4

Manuscript title: The implications of crustal architecture and transcrustal upflow zones on the metal endowment of a world-class mineral district

Caption: Figure S4. Geophysical data locations map. Yellow color represents MT sites, red and black colors represent gravity locations, and seismic profile. The Seequent software Geosoft Oasis Montaj (https://www.seequent.com/products-solutions/geosoft-oasis-montaj/) was used to generate the data location map.

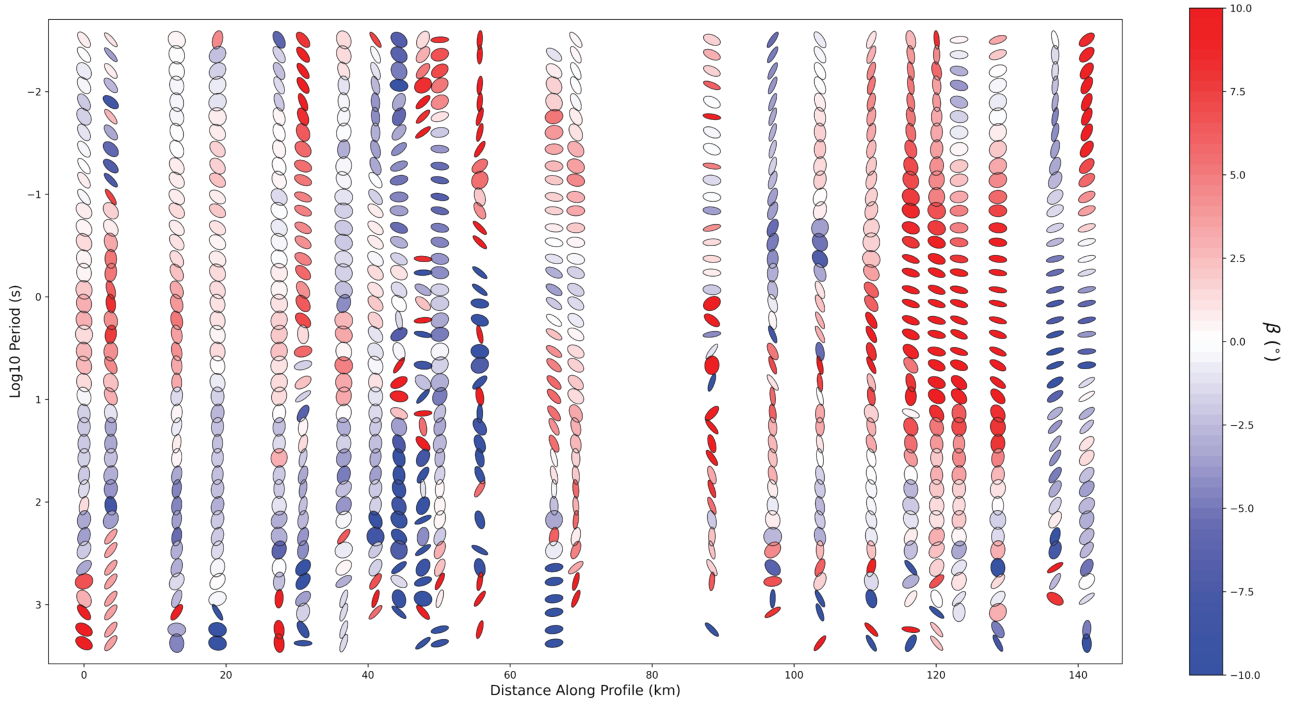

Author name: Taus R. C. Jørgensen et al.

Figure number: S5

Manuscript title: The implications of crustal architecture and transcrustal upflow zones on the metal endowment of a world-class mineral district

Caption: Figure S5. Phase tensor pseudosection along the route Noranda transect. Ellipses are coloured by β (a rotational invariant indicative of dimensionality).

Table S1. Geological features of the Noranda district.

Area	Ore (Mt)	Au (g/t)	Faults intersected by transect	Fault density (faults/km transect)	Felsic volcanic rocks	Mafic-intermediate volcanic rocks	TTG intrusions	Diorite and gabbro intrusions
1 - Northern Hunter block	4	<2	3	0.25	2	70	0	12
2 - Southern Hunter block	1	<2	4	0.19	18	54	2	14
3 - Flavrian block	24.5	<2	15	1.24	14	28	25	11
4 - Powell & Horne block	79.5	>5	7	1.65	25	24	21	14
5 - Rouyn-Palletier block	0	N/A	0	0.00	14	49	0	15

Table S2. Deposit location, tonnage, and grade

Name	Easting UTM NAD83 Zone N17	Northing UTM NAD83 Zone N17	Figure 1 number	Deposit type	Mt ore	%Cu	%Zn	Au (g/t)
Horne mine	647469.8	5346494.94	I	Au-rich Cu Zn-VMS	54.3	2.22		6.1
Quemnt mine	647908.3	5347219.42	II	Au-rich Cu Zn-VMS	16.65	1.2	1.8	5.5
Magusi River mine	620599.89	5366304.41	1	Cu Zn-VMS	3.73	1.2	3.55	1.1
Fabie Bay mine	622182.06	5366446.33	2	Cu Zn-VMS	0.89	2.59		0.9
Ansil mine	639891.4	5357276.28	3	Cu Zn-VMS	1.58	7.22	0.94	1.6
Vauze mine	642331.74	5357724.31	4	Cu Zn-VMS	0.35	2.9	0.94	0.7
Bouchard-Hebert mine	655744.07	5361270.43	5	Cu Zn-VMS	20.33	0.76	3.42	1.28
East Waite mine	642836.97	5356037.05	6	Cu Zn-VMS	1.5	4.1	3.25	1.8
Waite mine	641674	5355608.08	7	Cu Zn-VMS	1.12	4.7	2.98	1.1
Norbec mine	644629.09	5357142.83	8	Cu Zn-VMS	4.47	2.75	4.75	0.91
Amulet F mine	642455.67	5353348.86	9	Cu Zn-VMS	0.27	3.4	8.6	0.3
Gallen/West McDonald mine	651959.64	5354397.45	10	Cu Zn-VMS	8.1	0.08	3.36	0.06
Amulet C, A mine	642789.31	5352452.8	11	Cu Zn-VMS	5.9	4.65	5.4	1.31
Corbet mine	642465.2	5351337.49	12	Cu Zn-VMS	2.78	2.92	1.62	1
Millenbach mine	644381.25	5351509.08	13	Cu Zn-VMS	3.56	3.46	4.33	1
Aldermac	631559.94	5341871.65	14	Cu Zn-VMS	2.86	1.54	4.12	0.48
Deldona	651158.91	5347800.91	15	Cu Zn-VMS (Au-anomalous)	0.09	0.3	5	4.1
Delbridge mine	651034.98	5347314.74	16	Cu Zn-VMS (Au-anomalous)	0.36	0.55	8.6	2.4
McWatters mine	655448.56	5342024.17		Au-Orogenic	0.33			11.07
Lac Bouzan mineralization	649795.75	5340317.84		Au-Orogenic	-			
Astoria mine	646116.17	5339593.36		Au-Orogenic	0.35			~5
Lac Gamble mineralization	644524.23	5339478.97		Au-Orogenic	-			
Augmitto mineralization	641588.2	5339574.3		Au-Orogenic	-			
Cinderella mineralization	642655.85	5339402.71		Au-Orogenic	-			
Silidor mine	642884.63	5345932.52		Au-Orogenic	2.96			5.08
New Marlon mine	643580.51	5347295.68		Au-Orogenic	0.1			6.14
Powell-Rouyn mine	645000.86	5347133.63		Au-Orogenic	2.72			4.43
Anglo-Rouyn mine	644848.34	5348353.8		Au-Orogenic	0.13			8.6
Powell-Rouyn 2 mineralization	645725.34	5347819.97		Au-Orogenic	-			
Donalda mine	649748.08	5347152.69		Au-Orogenic	0.81			~5.6
Granada mine	646935.98	5337925.16		Au-Orogenic (syenite associated)	0.21			7.8
Beattie mine	630681.89	5374215.19		Au-Orogenic (syenite associated)	8.4			3.52
St-Jude Breccia mineralization	631699.99	5352151		Intrusion hosted Au +/- Cu +/- Mo +/- Ag	-			
Don Rouyn	643656.77	5345780		Intrusion hosted Au +/- Cu +/- Mo +/- Ag	5.18	0.15		

Data from Gibson & Galley (2007) and Système d'information géominière of Québec (SIGÉOM; https://sigeom.mines.gouv.qc.ca/signet/classes/I1102_aLaCarte?l=a#).

Table S3. Deposit distance (m) to 100 ohm*m contour at depths from ~1-40 km. Distances are set to 0 m where deposits fall within the 100 ohm*m contour.

	Au-rich VMS		Average	Au-anomalous VMS		Average
Depth (km)	Horne mine	Quemnt mine		Deldona	Delbridge mine	
1.14	0.00	0.00	0.00	0.00	0.00	0.00
1.41	0.00	0.00	0.00	0.00	0.37	0.18
1.73	0.00	0.00	0.00	0.35	0.48	0.41
2.10	0.00	0.00	0.00	1.24	1.13	1.19
2.53	1.34	2.18	1.76	1.58	1.54	1.56
3.03	2.85	3.68	3.27	1.66	1.64	1.65
3.62	3.39	4.21	3.80	1.71	1.68	1.70
4.30	3.83	4.62	4.23	2.60	2.65	2.63
5.10	4.78	5.52	5.15	2.58	2.74	2.66
6.03	5.37	5.89	5.63	2.61	2.77	2.69
7.11	7.17	6.64	6.91	3.36	3.52	3.44
8.38	7.77	7.19	7.48	3.89	4.12	4.01
9.85	8.75	8.18	8.47	4.88	5.09	4.99
11.60	9.13	8.62	8.88	5.35	5.50	5.43
13.60	8.89	8.44	8.67	5.25	5.32	5.29
15.90	8.62	8.17	8.40	4.98	5.05	5.02
18.60	7.19	6.78	6.99	3.61	3.66	3.64
21.80	0.00	0.00	0.00	0.00	0.00	0.00
25.50	0.00	0.00	0.00	0.00	0.00	0.00
29.80	0.48	0.00	0.24	0.00	0.58	0.29
34.80	3.28	3.04	3.16	5.44	5.54	5.49
40.70	5.76	6.10	5.93	9.32	9.21	9.27

							Cu-Zn VMS								Average
Depth (km)	Magusi River mine	Fabie Bay mine	Ansil mine	Vauze mine	Bouchard-Hebert mine	East Waite mine	Waite mine	Norbec mine	Amulet F mine	llen/West McDonald m	Amulet C, A mine	Corbet mine	Millenbach mine	Aldermac	
1.14	1.42	1.31	1.87	2.33	7.13	0.57	1.43	2.22	0.65	4.35	0.67	1.78	0.00	12.80	2.75
1.41	1.34	1.28	1.89	3.14	6.85	1.42	1.72	2.86	0.65	4.34	0.63	1.77	0.00	11.80	2.83
1.73	1.56	1.32	1.95	3.18	10.10	2.45	2.46	3.61	0.66	4.96	0.76	1.84	0.35	11.40	3.33
2.10	2.88	2.34	5.73	4.36	10.20	3.17	3.55	3.93	1.83	5.28	1.63	2.52	1.57	10.70	4.26
2.53	3.20	2.38	5.95	4.63	10.40	6.26	6.83	5.37	8.13	6.98	7.18	6.18	6.08	10.50	6.43
3.03	6.03	4.77	6.12	4.83	10.50	6.46	7.03	5.44	8.23	6.89	7.29	6.26	6.22	10.50	6.90
3.62	6.58	5.24	6.25	4.97	10.40	6.61	7.17	5.49	8.76	6.82	7.85	6.76	6.86	10.50	7.16
4.30	8.07	6.63	6.87	5.33	10.40	6.85	7.54	5.64	8.91	6.67	8.00	6.91	7.16	10.50	7.53
5.10	10.70	9.17	7.57	6.20	9.96	7.70	8.41	6.47	9.06	6.24	8.14	7.05	7.31	10.50	8.18
6.03	9.70	9.45	8.02	6.56	8.81	8.08	8.77	6.84	10.00	6.19	9.09	7.99	8.24	10.30	8.43
7.11	8.58	8.82	8.91	7.55	7.89	9.10	9.77	7.85	11.80	6.01	12.30	12.30	10.50	10.90	9.45
8.38	8.22	8.35	9.97	8.68	6.29	10.10	10.90	8.48	12.80	6.19	12.80	12.80	11.00	10.10	9.76
9.85	7.88	7.77	9.99	8.73	3.89	9.99	10.90	8.35	12.60	6.73	13.40	13.80	12.00	9.05	9.65
11.60	8.00	7.51	10.30	8.77	0.00	10.00	10.90	8.36	12.70	6.59	13.40	14.50	12.70	7.71	9.39
13.60	9.13	8.14	11.00	8.94	0.00	9.79	10.90	7.75	12.00	4.58	12.50	13.50	12.10	6.27	9.04
15.90	13.80	12.60	11.10	8.91	0.00	9.54	10.70	7.46	11.60	3.66	11.90	12.30	11.30	5.07	9.28
18.60	21.10	19.80	11.90	9.60	0.00	10.00	11.20	7.93	11.20	3.36	10.50	9.30	10.20	3.84	10.00
21.80	6.78	5.38	6.56	8.61	0.00	7.77	6.64	9.33	5.63	2.24	5.19	4.03	4.18	2.92	5.38
25.50	9.37	8.13	0.48	2.14	5.09	0.94	0.00	2.49	0.00	0.00	0.00	0.00	0.00	4.92	2.40
29.80	15.40	14.30	0.51	1.48	8.58	0.00	0.00	1.31	0.00	0.74	0.00	0.00	0.00	8.06	3.60
34.80	17.60	19.00	3.93	4.34	13.60	2.79	2.21	4.13	0.56	6.64	0.00	0.00	0.00	9.12	5.99
40.70	15.80	17.10	8.03	8.59	19.00	7.08	6.40	8.78	4.43	11.90	3.77	2.66	4.08	0.00	8.40

							Orogenic Au								Average
Depth (km)	McWatters mine	Lac Bouzan mineralization	Astoria mine	Lac Gamble mineralization	Augmitto mineralization	Cinderella mineralization	Silidor mine	New Marlon mine	Powell-Rouyn mine	Anglo-Rouyn mine	Powell-Rouyn 2	Donalda mine	Granada mine	Beattie mine	
1.14	4.47	0.82	4.15	4.74	5.34	5.08	1.42	1.43	0.48	1.45	0.80	0.99	2.49	11.30	3.21
1.41	4.21	1.44	4.60	4.56	4.91	4.86	1.36	1.40	0.78	1.85	1.03	1.43	3.37	8.57	3.17
1.73	3.30	1.96	4.14	3.80	3.89	3.85	1.22	1.61	0.96	2.18	1.38	1.59	3.36	8.23	2.96
2.10	3.75	2.34	4.00	3.75	3.73	3.72	1.07	1.85	1.59	2.66	1.77	1.80	3.37	8.32	3.12
2.53	3.88	5.53	3.94	3.25	3.21	3.03	1.06	2.00	1.84	2.97	2.55	2.80	5.80	8.52	3.60
3.03	4.09	5.70	2.44	1.53	2.72	2.18	1.25	2.07	1.95	3.11	2.84	2.90	2.62	5.74	2.94
3.62	4.39	6.08	2.82	2.05	2.72	2.56	1.34	2.73	2.58	3.75	3.48	2.94	2.66	4.64	3.20
4.30	4.79	6.22	3.64	2.99	2.74	2.74	1.49	2.88	2.94	4.11	3.81	3.92	3.02	4.29	3.54
5.10	5.15	6.62	3.82	2.85	2.72	2.58	1.63	3.02	3.37	4.40	4.34	4.04	3.40	5.20	3.80
6.03	5.56	6.93	3.94	3.01	2.51	2.77	2.57	3.96	4.22	5.28	5.17	4.07	4.06	5.25	4.24
7.11	5.61	8.75	5.76	4.76	4.09	3.98	10.50	10.90	9.54	9.61	8.76	4.81	5.02	5.83	6.99
8.38	5.65	9.41	6.31	5.26	4.14	4.32	10.60	11.50	10.10	10.10	9.28	5.40	5.88	6.71	7.48
9.85	5.66	9.78	7.65	6.32	4.62	5.02	11.00	12.50	11.10	11.10	10.30	6.38	7.41	5.90	8.20
11.60	5.17	9.82	8.17	6.78	4.60	5.22	10.70	12.20	11.50	11.70	10.80	6.79	8.19	1.94	8.11
13.60	4.46	9.34	7.54	6.05	3.61	4.41	9.33	10.90	11.30	11.60	10.70	6.60	7.87	0.00	7.41
15.90	3.72	8.46	6.54	4.95	2.18	3.11	7.60	9.13	9.77	10.70	10.40	6.33	7.13	1.33	6.53
18.60	2.22	6.97	4.87	3.27	0.41	1.40	4.71	6.22	7.03	7.83	8.03	4.94	5.85	8.29	5.15
21.80	0.00	0.00	0.00	0.00	0.00	0.00	0.00	0.48	0.00	1.09	0.41	0.00	1.54	10.10	0.97
25.50	0.47	1.41	2.09	2.51	3.20	2.98	0.00	0.00	0.00	0.00	0.00	0.00	3.68	12.50	2.06
29.80	6.85	6.59	7.02	7.14	7.26	7.31	0.79	0.00	0.00	0.00	0.00	0.00	8.69	17.40	4.93
34.80	12.20	9.76	9.01	8.72	8.33	8.53	2.16	0.92	1.46	0.41	1.31	4.55	10.80	22.50	7.19
40.70	14.60	10.20	7.70	6.77	5.14	5.80	1.52	1.81	3.23	3.07	3.89	7.94	9.48	22.80	7.43
